# Twin Research and Imaging

**DOI:** 10.3390/medicina58060805

**Published:** 2022-06-15

**Authors:** David Laszlo Tarnoki, Adam Domonkos Tarnoki

**Affiliations:** Medical Imaging Centre, Hungarian Twin Registry, Semmelweis University, 78/A Üllői Street, 1082 Budapest, Hungary; tarnoki2@gmail.com

Twins have been the focus of scientific research for more than a century. Identical twins originate from one egg cell that splits in two at a very early stage of development, whereas non-identical twins are formed when two fertilised eggs implant in the womb at the same time, therefore, monozygotic twins (MZ) share almost 100% of their genes, whereas dizygotic twins (DZ) share 50% of their genes in average [1]. Twins are special subjects of research, as they help to tease apart the effects of nature (genetics) and nurture (environment) on human health.

The classical twin study design relies on studying MZ and DZ twins, which estimates the variance of genetic, shared and unique environmental factors in various phenotypes and diseases [2].

Other important study design is research of identical twins discordant for chronic diseases. In this model, the healthy co-twin serves as a control. The growing number of twin registries worldwide provides a unique resource for clinical, epidemiological, and genetic studies. Twin studies require complex statistical software during the data analysis. Twin studies will continue to be an important tool along with emerging genome and molecular research methods in shedding light on various aspects of human genetics and on how environmental factors, developmental variation and genetics combine to create human traits and behaviours [3].

There are many different types of medical imaging techniques, which use different technologies to produce images for different purposes. The main imaging techniques include radiography, ultrasound, computed tomography, magnetic resonance imaging, mammography, and nuclear medicine. The number of innovations is growing, and new imaging techniques are being developed. Imaging studies can be combined with twin research as well, mostly in discordant patient–control samples, which have revealed significant additive genetic influences on some chronic diseases. In the past decade, the number of prospective imaging studies is rising, using mostly non-ionizing imaging modalities, e.g., magnetic resonance imaging (MRI) or ultrasound in twins. Low dose computed tomography (CT) imaging provides a unique tool to visualize certain lung diseases or calcified coronary atherosclerosis, which is not visible without X-ray radiation. Imaging has had a rapid development in the last years since artificial intelligence (AI) became one of the most useful tools in radiology. AI will play an increasingly significant role in medical imaging and will become standard in the workflows of radiologists [4].

Research papers in the fields of radiology and/or twin studies are welcome in the current Special Issue entitled “Twin research and imaging”.

## Data Availability

Not applicable.
